# The additive value of 3D automated breast ultrasound and contrast enhanced spectral mammography in the assessment of suspicious breast lesions

**DOI:** 10.1186/s43046-026-00357-8

**Published:** 2026-05-26

**Authors:** Lamiaa Hassan ELmalatawy, Noha Abd El- Shafy Elsaid

**Affiliations:** Department of Radio-Diagnosis, National Cancer institute & Baheya Foundation, Cairo, Egypt

**Keywords:** Breast cancer, Automated breast ultrasound (ABUS), Contrast enhanced spectral mammography (CESM) and suspicious breast lesion

## Abstract

**Background:**

Worldwide, breast cancer is the most commonly diagnosed cancer among women accounting for over a million cases each year. Mammography still remains the gold standard for early detection of breast cancer. Contrast Enhanced Spectral Mammography (CESM) allows better estimation of the lesion size and detection of multifocal breast cancers. Automated breast ultrasound (ABUS) is an innovation of breast ultrasound that attains 3-D images of the entire breast overcoming the limitations of hand-held ultrasound. The aim is to evaluate the impact of 3D ABUS in correlation with CESM on the characterization and assessment of disease extent.

**Design & population:**

The study was performed at the Female Imaging Unit of the National Cancer Institute (NCI), Cairo University and in Baheya Foundation. One hundred and six patients were randomly chosen out of patients who were initially given BIRADS IV – V by standard mammography. The contrast enhanced digital mammography and 3D automated ultrasound data were evaluated by two experienced radiologists; both observers were blinded to the pathological data of each patient.

**Results:**

Regarding primary outcome of our study in assessment of tumor size, extension and multiplicity, the use of ABUS after CESM showed significant increase in the sensitivity of the test (80% for combined test versus 40% for CESM alone), while it showed almost no significant difference in specificity (70% for CESM and Combined tests).The ABUS also showed more accuracy in detection of suspicious lesions (61%) than the CESM (40%.)

**Conclusion:**

ABUS is considered a revolution in breast scanning by ultrasound imaging that can be used as a non-invasive complementary tool to CESM in assessment of suspicious breast lesions showing increased accuracy of study and patient outcome upon combing both of them.

## Introduction

Breast cancer is one of the leading causes of cancer related mortality in women worldwide and the second leading cause of death after lung cancer [1].

Once a diagnosis of breast cancer is established, the treatment planning depends on several factors including the patient’s age, the size of the tumor, the initial extent of disease and the different biologic characteristics of the tumor [2]. Mammography actually stays the highest quality level for early identification of breast disease, however different strategies were invented, for example, contrast-enhanced spectral mammography (CESM) and automated breast ultrasound (ABUS) to improve diagnosis and conclusion of different breast diseases [3].

Contrast Enhanced Spectral Mammography (CESM) improves assessment of the lesion size and detection of multifocal breast cancers than mammography alone or combined with ultrasonography. However, it carries the hazards of exposure to high doses of radiation and risks associated with contrast injection [4, 5]. On the other hand, Automated breast ultrasound (ABUS) is an innovation of breast ultrasound that comfortably and quickly attains 3-D images of the entire breast overcoming the limitations of hand-held ultrasound (HHUS), independent of the operator, more easily reproducible, and having a large probe that provides whole coverage of the breast and proper ability for characterization of large masses [6]. In the field of oncologic surgery, an accurate preoperative assessment of tumor location and extension are considered of key importance to guide treatment decisions and avoid recurrent disease, so that in our study, we aimed to evaluate the impact of 3D ABUS in correlation with CESM on the characterization and assessment of disease extent as regards to tumor size, extension and multiplicity in patients with suspicious breast lesions.

## Patients and methods

### Design and population

The study was performed at the Female Imaging Unit of the National Cancer Institute (N.C.I), Cairo University and in Baheya Foundation, during the period from July 2018 to December 2021. One hundred and twenty-three patients were randomly chosen out of patients who were initially given BIRADS IV – V by standard mammography (inclusion criteria).

All patients have offered full history taking, the three radiological modalities (standard mammography, CESM and ABUS) and obtained biopsies by experienced staff.

Exclusion criteria included Patients with severe renal impairment or known allergy to the contrast, early post-operative cases or recently treated by radiotherapy (to minimize the false positive results with CESM), patients with poor general condition and pregnant women.

### Statistical analysis

Data were coded and entered using the statistical package SPSS (Statistical Package for the Social Sciences) version 24. Data was summarized using mean, standard deviation, median, minimum and maximum in quantitative data and using frequency (count) and relative frequency (percentage) for categorical data. For comparison of paired measurements to lesion size, the non-parametric Wilcoxon signed rank test was used. For comparing categorical data, Chi square test was performed. Exact test was used instead when the expected frequency is less than 5 [7]. Standard diagnostic indices including sensitivity, specificity, positive predictive value (PPV), negative predictive value (NPV) and diagnostic efficacy were calculated as described by [7]. Agreement between different methods in categorical variables was done using Kappa measure of agreement. As a measure of reliability Kappa should be greater than 0.70 (70%) not only to be significant, but Kappa more than 0.40 is to be considered fair. Correlations between quantitative variables were done using Spearman correlation coefficient thus, P-values less than 0.05 were considered as statistically significant.

## Results

**Fig. 1:**
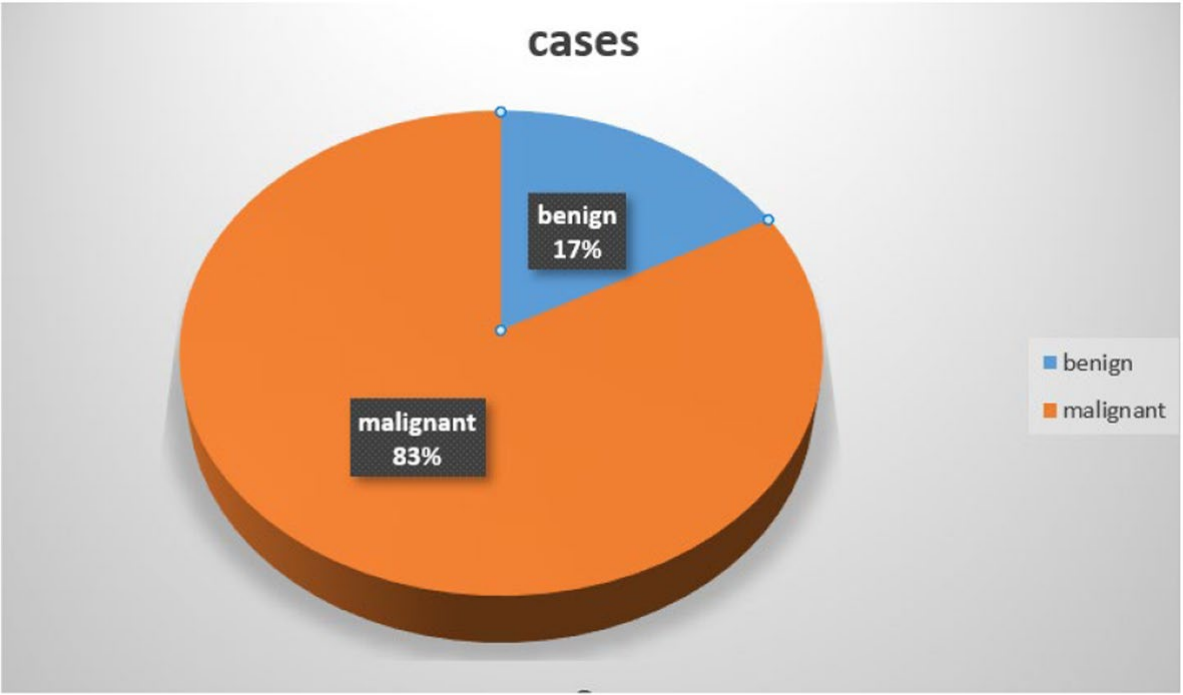
Demonstrate ratio between malignant and benign lesions malignant lesions represent (83%) while benign lesions represent (17%) of patients

Seventeen out of the total number of patients were excluded due to switch to neo-adjuvant chemotherapy after completing the study imaging procedures, the data had been lost or patients were not coming back for follow up. Eighty-eight patients were diagnosed as malignant breast lesions and they were approached for treatment according to the protocol, while eighteen patients were diagnosed as benign breast lesions recommended to follow up (as illustrated in Fig. 1).

ABUS shows more sensitivity (95.65%) in detection of ductal extension while CESM was more specific (100%) with total accuracy rate in favor of ABUS as shown in Table 1.

**Table 1:** Showed detailed analysis of Combined results of both modalities in detection of ductal extension

Measure	CESM	ABUS	Derivations
Sensitivity	63.64%	95.65%	TPR = TP/(TP + FN)
Specificity	100%	98.46%	SPC = TN/(FP + TN)
positive predictive value	100%	95.65%	PPV = TP/(TP + FP)
Negative Predictive Value	89.19%	98.46%	NPV = TN/(TN + FN)
Accuracy	90.91%	97.73%	ACC = (TP + TN)/(P + N)

The most sensitive technique in detecting tumor multiplicity was CESM (97.83%), while the most specific was ABUS (97.67%), with improved diagnostic performance and increased total accuracy rate using combined CESM&ABUS (97.75%) as explained in Table 2.

**Table 2:** Detailed analysis of results of Combined both CESM and ABUS in detection multiplicity

Measure	ABUS	CESM	Addedvalueboth CESM and ABUS
Sensitivity	93.33%	97.83%	97.83%
Specificity	97.67%	92.86%	97.67%
positive predictive value	97.67%	93.75%	97.83%
Negative Predictive Value	93.33%	97.50%	97.67%
Accuracy	95.45%	95.45%	97.75%

**Fig. 2:**
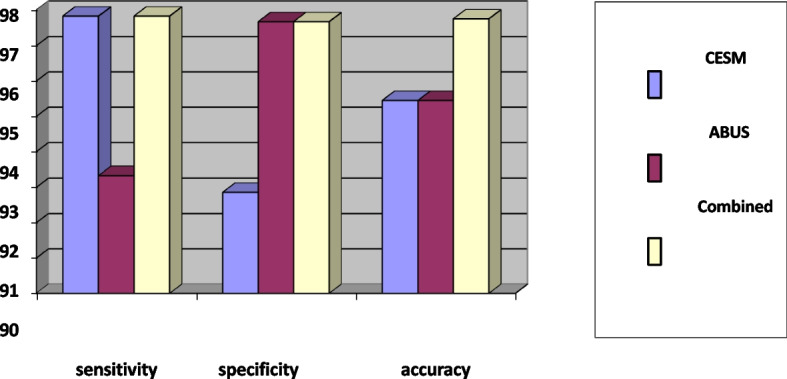
Accuracy of each study alone (95.5%) while combining both modalities successfully rising up accuracy to approximately (98%)

Figure 2 demonstrates Comparison between impact of ABUS/CESM Versus combined both modalities regarding the sensitivity, specificity and accuracy.

**Fig. 3:**
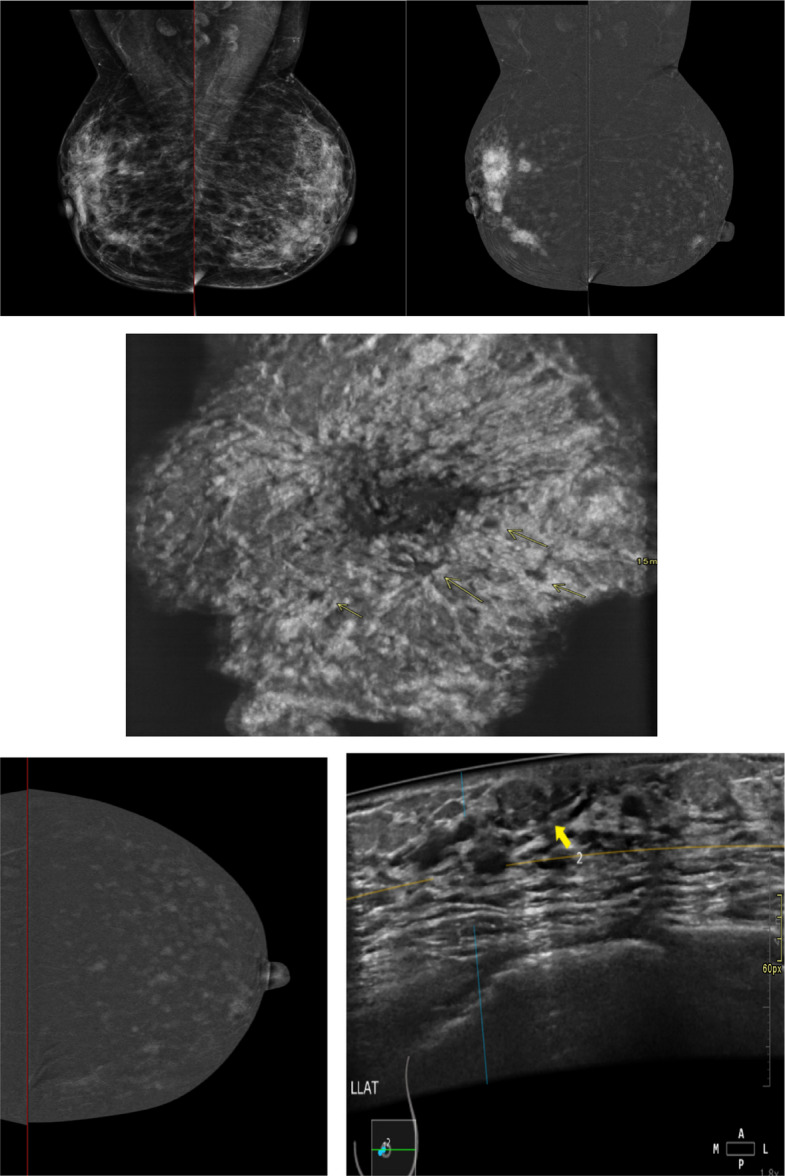
45 year old female complaining of right palpable lump and retracted nipple. DM: Bilateral heterogenous parenchyma with right retracted nipple and asymmetry. CESM: marked background enhancement with: right clumped intense mass and non-mass enhancement -left peri-areolar intense enhancing lesion. Case shown pathologically proven invasive lobular carcinoma (BIRADS-6)

**Fig. 4:**
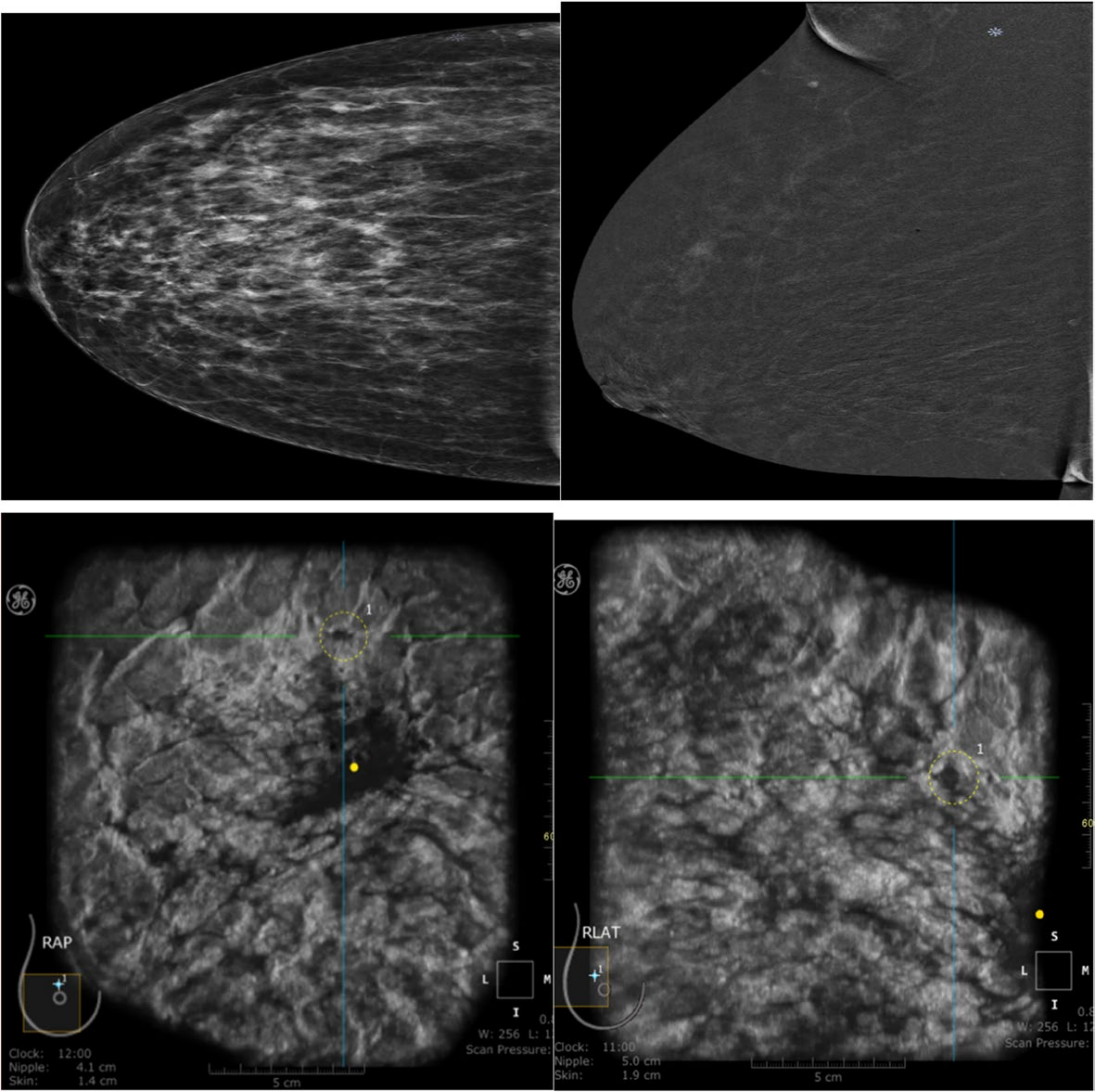
55 year old was coming for routine screening, Digital mammography showing right heterogeneous fibroglandular tissue (ACR C). Complementary ABUS depicted small spiculated hypoechoic defect in coronal view corresponding to small hypoechoic lesion with spiculated margins on axial views; CESM showed corresponding heterogeneous mass enhancement -case shown pathologically proven IDC G1, (BIRADS-6)

**Fig. 5:**
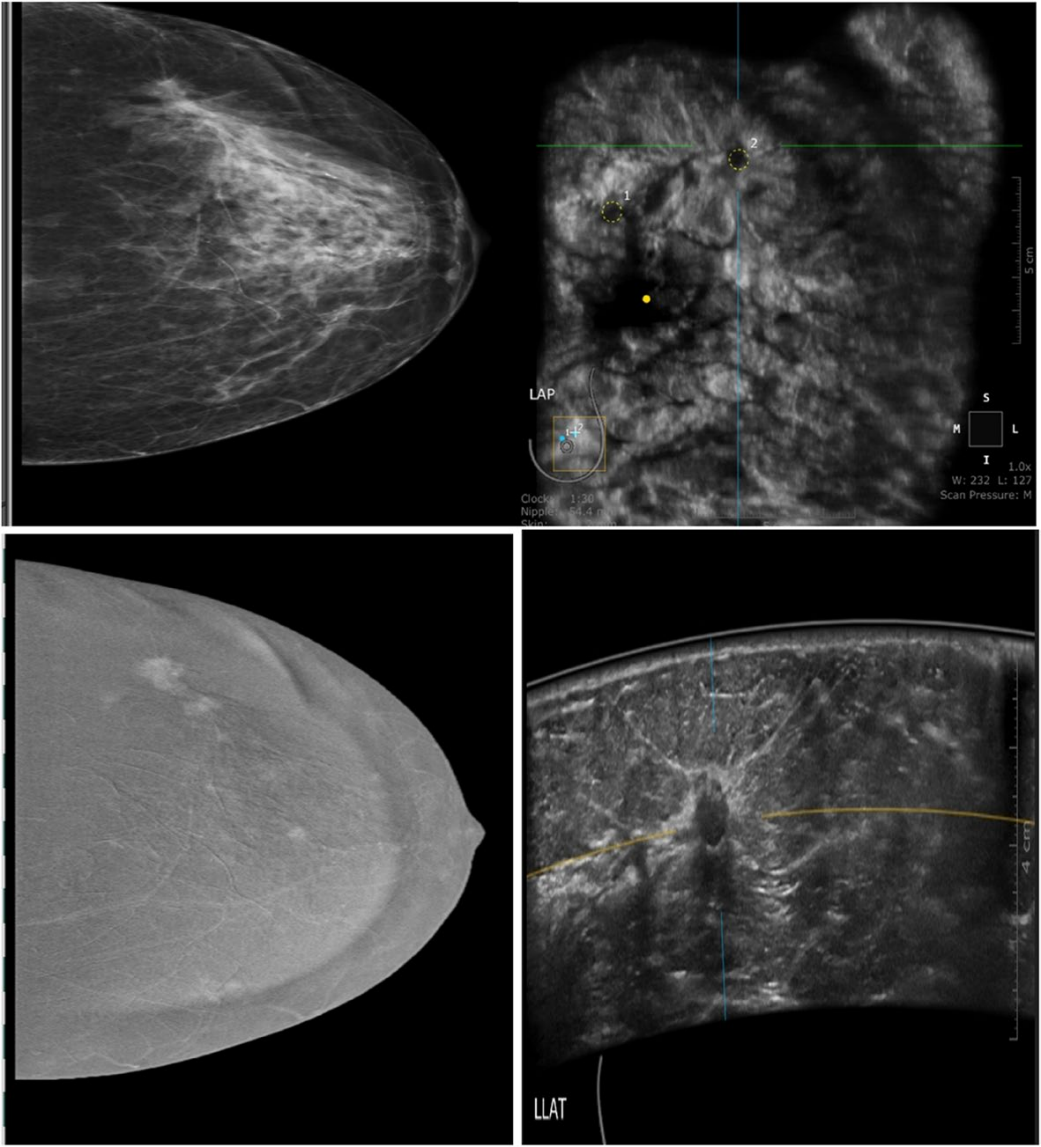
60 year old with left palpable lump multiple heterogeneous enhancing Lesions with most anterior lesion located about 2 cm from NAC. ABUS coronal view: Proper delineation of ductal extension (blue arrows). Case shown Histopathology confirmed IDC with lobular component (BIRADS-6)

**Fig. 6:**
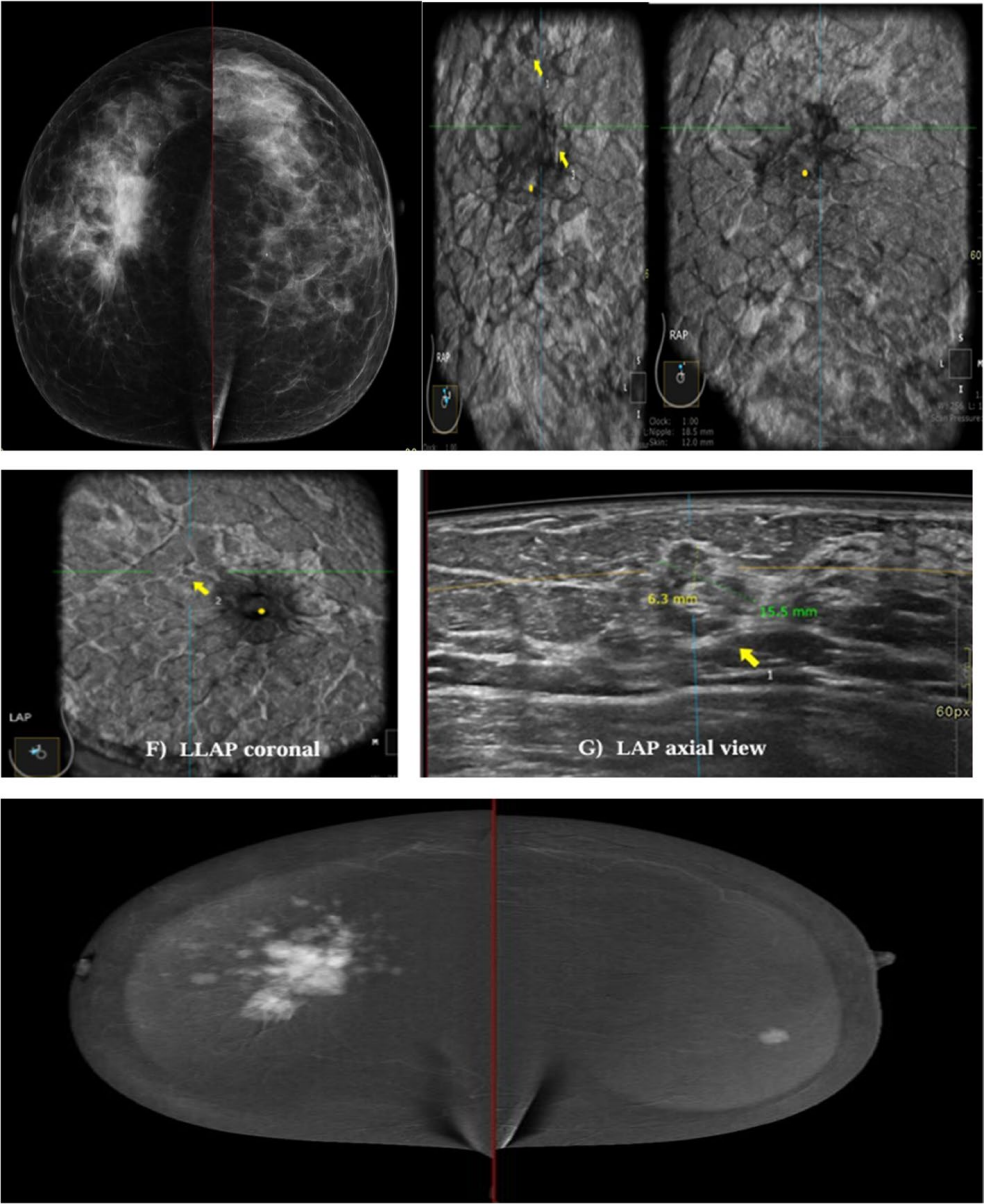
55 year old with high suspected lesion with digital mammography: showed right spiculated lesion and dense irregular asymmetry corresponding to right multiple hypoechoic irregular defects on ABUS, as well as noticed left small lesion with subtle margin irregularity. Left intense homogenous enhancing lesion (benign mimics). Case shown pathologically proven right IDC and left mucinous carcinoma (BIRADS-6)

The ABUS was able to significantly downgrading BIRADS in benign lesions (as shown in Table 3) 14 cases could be downgraded from IV-III, and correctly upgrading the BIRADS (18 cases as shown in Table 4) in malignant lesions (Figs. 3, 4, 5 and 6).

**Table 3:**
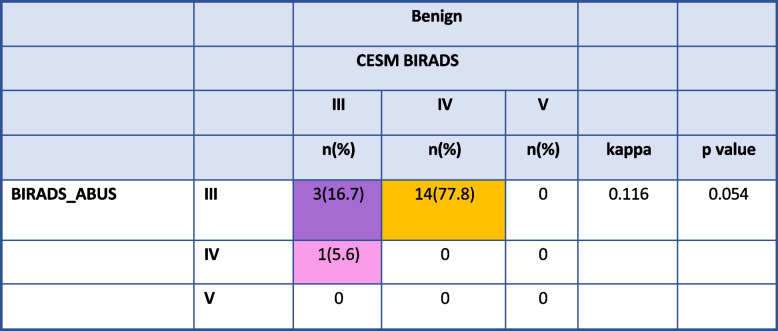
The BIRADS re-reporting after CESM & ABUS for benign lesions

**Table 4:**
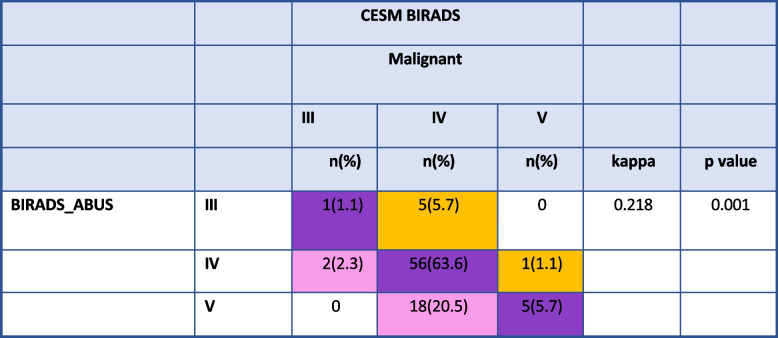
The BIRADS re-reporting after CESM & ABUS for malignant lesions

## Discussion

Breast mammography is a well-known, cost-effective imaging technique for early detection of breast cancer which has shown to reduce mortality. However, it has low sensitivity and specificity in dense and treated breasts due to the decreased contrast between a probable tumor and the surrounding breast tissue [2]. Moreover, Breast ultrasound is considered a valuable complementary technique to mammography with a definite role in detection and characterization of breast lesions. And, it is established that using breast ultrasound improves breast cancer detection when compared to mammography alone [3]. Our study was concerned of new modalities in evaluation of suspicious breast masses and if the combined use may enhance the accuracy of lesions detection.

It has been suggested that CESM is a reliable tool to generate good diagnostic accuracy in characterizing benign from malignant breast lesions, but with few exceptions [8]. Furthermore, ABUS is a promising new technology that had been FDA- approved for screening, it may also be useful for characterization, diagnosis, staging, and follow up of management [9, 10].

Regarding the *ductal extension* of the tumor *(stromal invasion),* our study confirmed the ability of CESM to detect malignant breast tumors in either one or both breasts. The results also showed a higher sensitivity of CESM as well as better specificity when compared to DM alone or in combination with automated ultrasound. However, in our data CESM showed higher specificity alone, and ABUS showed higher sensitivity with more accuracy of ABUS (97.7%) in detection of ductal extension compared to CESM (90.9%).

The ABUS also was able to correctly upgrading the BIRADS in malignant lesion and downgrading the BIRADS in benign lesions in comparison to CESM results.

This advantage showed the additive value of ABUS to improve the accuracy of diagnosis of different breast lesions. Regarding *size* agreement, all lesions in our study were detected by the two practitioners using ABUS which led to increased sensitivity for the detection of breast cancer and showed high reliability of this imaging tool. Our study reported also a higher rate of CESM in under-estimating size of the lesion in comparison to pathology results. The difference in size estimation between CESM, ABUS, and pathology report increased with increasing lesion size might be due to the fact that appropriate evaluation of the extent of a lesion in pathology becomes more difficult when the pathologist evaluates a lesion that is presenting in multiple slices of a specimen [11].

Furthermore, regarding *multiplicity* of the lesion, CESM was the most sensitive imaging modality in detecting tumor multiplicity in this study (97.8%) followed by ABUS (93.3%) and was less specific (92.8%) than ABUS (97.67%) with increased total accuracy upon combining both modalities reaching 97.7% compared to 95.4%.

### Study limitations

This study has some limitations including the relatively limited sample size, Limited ability of proper assessment of lymph nodes with ABUS, which can be minimized by applying extended axillary view, yet still restricted to level I axillary nodes only and Back ground enhancement in CESM resulting in false positive results requiring additional work up, to minimize this risk CESM best performed in the luteal phase of menstrual cycle. Moreover, larger multicenter studies are recommended to further evaluate the diagnostic performance of combined CESM and ABUS in the assessment of suspicious breast lesions.

### Clinical implications

The combined use of CESM and ABUS may provide additional diagnostic information in patients with suspicious breast lesions, particularly in women with dense breast tissue. This combined approach will improve preoperative assessment of tumor extent and assist in treatment planning.

## Conclusion and recommendations

The study showed that ABUS can detect mammographically occult breast lesions and accurately detects lesion location and size. Furthermore, accuracy of each study alone (95.5%), while Combining both modalities successfully raising up accuracy in detection of breast lesions to approximately (98%). Obviously, ABUS is considered a revolution in breast scanning by ultrasound imaging that can be used as a non-invasive alternative tool to CESM in the assessment of breast cancer. We can then recommend the use of ABUS as an adjunct to CESM in the early detection of breast cancer.

## Data Availability

The datasets used and/or analyzed during the current study are available from the corresponding author upon request.
